# High Frequency MEMS Capacitive Mirror for Space Applications

**DOI:** 10.3390/mi14010158

**Published:** 2023-01-08

**Authors:** Alvise Bagolini, Anze Sitar, Luca Porcelli, Maurizio Boscardin, Simone Dell’Agnello, Giovanni Delle Monache

**Affiliations:** 1 Center for Sensors and Devices (SD), Fondazione Bruno Kessler (FBK), Via Sommarive 18, 38123 Trento, Italy; 2 Istituto Nazionale di Fisica Nucleare-Laboratori Nazionali di Frascati (INFN-LNF), Via E. Fermi 40, 00044 Frascati, Italy; 3 Dipartimento di Fisica, Università della Calabria (Unical), Via Pietro Bucci, Arcavacata, 87036 Rende, Italy

**Keywords:** micromirrors, MEMS, free space optics

## Abstract

Free space optics laser communication using modulating retroreflectors (MR) is a challenging application for an active mirror, due to the high frequencies (>100 kHz) required to enable sufficient data transfer. Micro Electromechanical (MEMS) mirrors are a promising option for high-frequency applications, given the very small moving mass typical of such devices. Capacitive MEMS mirrors are presented here for free space communications, based on a novel fabrication sequence that introduces a single-layer thin film aluminum mirror structure with an underlying silicon oxide sacrificial layer. The use of aluminum instead of gold as a mirror layer diminishes the heating generated by the absorption of the sun’s radiation once the mirrors exit the earth’s atmosphere. Thanks to the novel fabrication sequence, the presented mirror devices have a full range actuation voltage of less than 40 V, and a high operational frequency with an eigenfrequency above 2 MHz. The devices were manufactured and characterized, and their main parameters were obtained from experimental data combined with finite element analysis, thus enabling future design optimization of the reported MEMS technology. By optical characterization of the far field diffraction pattern, good mirror performance was demonstrated.

## 1. Introduction

In the last decade, different approaches to micro electromechanical (MEMS) mirrors have been pursued, mainly depending on the actuation system. Piezoelectric [1], capacitive [2], magnetic [3,4], and thermomechanical [5] actuation mirrors were developed, and their use was demonstrated in a plethora of applications.

Given the very small mass typical of MEMS devices, these mirrors are a promising option for high frequency applications. Indeed, commercial devices with magnetic actuation [4] can operate at up to 100 Hz. Further, a 6.7 kHz MEMS mirror working at its resonance frequency was recently demonstrated by Seo [6].

Free space optics laser communication using modulating retroreflectors (MR) is a challenging application for an active mirror, due to the high frequencies (>100 kHz) required to enable sufficient data transfer. This suggests that MEMS mirrors are an ideal candidate for this application. It is noteworthy that, besides free space optics MR, such high-frequency MEMS mirrors could be suitable for a series of applications, including ground-to-air communications, ground-to-satellite communications, internal electronics bus interaction/communication, inter and intra-office communications, vehicle-to-vehicle communications, and industrial manufacturing.

The standard configuration for an intensity modulation retroreflector mirror is that of a hollow corner cube, where three mirrors are connected to create a solid cube corner. The incoming light beam will be reflected on each of the three mirrors and directed backward to its source. If one of the mirrors is capable of intensity modulation, the reflected beam will be modulated.

Boston Micromachines developed a prototype mobile MR MEMS mirror for free space optics with a frequency of 180 kHz [7]. They used a simple structure that enables the bending of portions of continuous mirrors [8] and has a high resonance frequency (>200 kHz). This resonance frequency represents a major achievement that is made possible using surface micromachined deformable thin film mirrors. As a comparison, the typical resonance frequency of rigid, bulk micromachined mirrors for emerging LiDAR applications, where the device is operated using torsional supports, is >0.8 kHz [9].

In the framework of the GLAREX project [10], a capacitive MEMS mirror is proposed here for free space communications at very high signal frequencies based on the approach proposed by Boston Micromachines. The mirror consists of a matrix of circular membranes that can be simultaneously deformed by applying a DC signal between the mirror layer and the silicon substrate.

The present mirrors introduce a novel single-layer thin film aluminum mirror structure combined with a silicon oxide sacrificial layer. The devices are designed to provide very high frequencies, switching in the MHz range, with low full-range actuation voltage.

Compared to a previous article [8] the use of aluminum instead of gold as a mirror layer prevents heating generated by the absorption of solar radiation once the mirrors exit the earth’s atmosphere, enabling the use of the device in space applications.

Further, the actuation potential is reduced by eliminating the highly stressed silicon nitride layer from under the mirror metal layer, compared to the 100 V reported in the previous article [8]. This, again, is a key enabler for space applications, as voltage onboard space vehicles, such as rovers, is typically limited to 28 V [11]. The membranes have a diameter between 60 and 100 μm which was determined by preliminary finite element modeling as a compromise between low voltage operation and high planarity.

We report the modeling, designs, simulations, and fabrication of two MEMS mirror devices. Further, we report the results of a series of preliminary electrical and optical tests addressing space application performance.

## 2. Materials and Methods

### 2.1. Design

Circular mirrors in a hexagonal arrangement were chosen to maximize the ratio between the active (dynamic) and the passive (static) surface of the mirror matrix.

**Figure 1:**
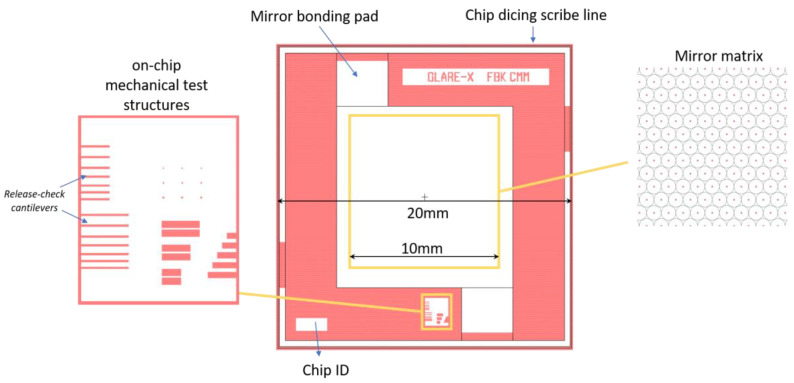
Device layout overview.

Holes at the center of each mirror enable its release by isotropic etching of the supporting sacrificial layer. The device size is 20 × 20 mm whereas each mirror matrix has a side of 10 mm and is made of circular membranes with a pitch from 60 to 100 μm, as seen in Figure 1. The radius of each circular membrane depends on the release step, as will be further detailed. The radius and pitch are determined with a preliminary simulation of the mirror electrostatic deformation, to enable low-voltage operation. A larger radius would allow for even lower voltages but was rejected to avoid possible loss of planarity due to large, suspended membranes being more prone to out-of-plane bending induced by residual stress.

### 2.2. Microfabrication

**Figure 2:**
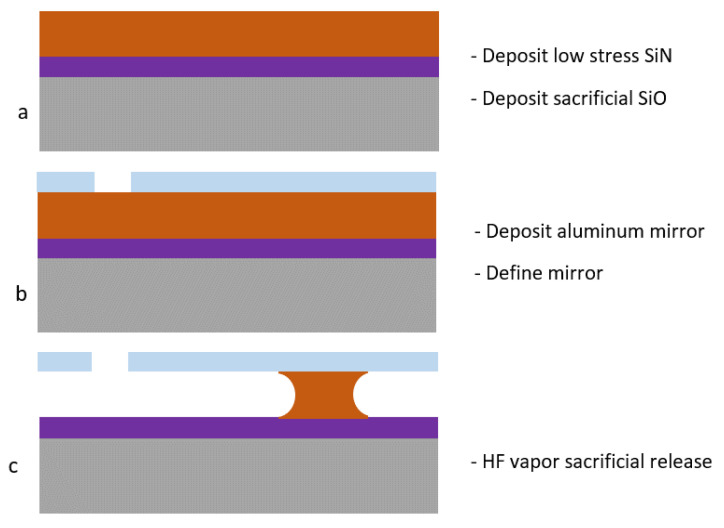
MEMS mirror fabrication sequence. Deposition of low stress silicon nitride and sacrificial silicon oxide (**a**), deposition and patterning of the mirror aluminum film (**b**), HF sacrificial silicon oxide removal to release the suspended structure (**c**).

The microfabrication was performed on SEMI standard 6″ silicon wafers, using standard IC technology equipment in a class 100 microfabrication cleanroom at FBK, Italy. The process has a single lithography step. First, a low stress silicon nitride (SiN) was deposited on the wafers by the LPCVD technique in a E1200HT Centrotherm furnace. Then, the sacrificial TEOS silicon oxide (SiO) was deposited by LPCVD again with a E1200HT Centrotherm furnace (Figure 2a). These layers were removed from the wafer backside to enable bulk silicon contact. Then the mirror aluminum layer was deposited by magnetron PVD on the wafer frontside using an MRC Eclipse tool, and another aluminum deposition was performed on the wafer backside to provide electrical contact. The front aluminum layer was patterned using a standard photolithography technique with an MA150 Suss mask-aligner and removed with dry etching in a TEGAL 6520 tool (Figure 2b). The mirror was released using HF vapor in an SPTS HFV etcher (Figure 2c).

**Figure 3:**
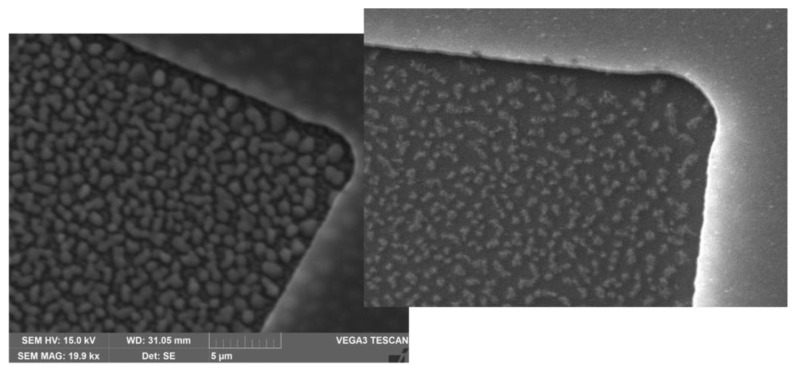
Residuals after HF vapor etching (**left**) and the effect of baking at 150 °C on the HF vapor residuals (**right**).

After etching, a bake was performed in an air convection oven, to reduce the size of residuals generated by HF vapor etching. Residuals appear as flakes of organic material generated by the combination of fluorine and carbon brought into the reaction chamber by alcohol used as a catalyst for the etching reaction (Figure 3, left). The suggested bake temperature to fully eliminate the etching residuals after HF vapor etching is 250 °C, but this temperature is not compatible with the aluminum mirrors, as it causes deformation of the suspended aluminum film. The best compromise temperature was found to be 150 °C with a 1 h bake time. This resulted in a considerable deflation of the residuals, without compromising the mirror integrity (Figure 3, right). Indeed, small flakes can remain on the surface of silicon nitride as long as they do not compromise the mirror actuation. An assessment of the effect of residuals on mirror actuation is reported in the characterization.

### 2.3. Morphological Characterization

After fabrication, interferometer measurements of the silicon nitride layer thickness were performed in the open areas outside the mirrors, to assess the final nitride thickness after the HF vapor release. By knowing the final thickness of the silicon nitride, it is possible to obtain the effective thickness of HF vapor residuals by applying a load on a dedicated suspended bridge structure (mechanical test structures reported in Figure 1) and measuring its downward bending. The tested aluminum bridge was 350 μm long and 50 μm wide with the same thickness as the aluminum mirrors. A KLA-Tencor profilometer was used, and a load of 5 mg was applied on the stylus to grant a complete deflection of the bridge structure and make it touch the underlying residuals. Lastly, the radii of the fabricated membranes were measured using optical images obtained with a microscope, using a reduced diaphragm aperture to have grazing illumination of the mirror surface.

### 2.4. Residual Stress Measurement

In order to enable the finite element simulation, the mirror’s aluminum residual stress is paramount data. The stress was directly measured by the wafer curvature method [12] on dedicated test wafers, both just after deposition and after the thermal budget caused by the subsequent microfabrication steps. A process equivalent thermal budget was obtained by baking in a convection oven with a nitrogen atmosphere at 140 °C for 60 min.

### 2.5. Electrostatic Actuation

To provide a complete description of the mirror deformation as a function of the applied voltage, and to assess the impact of HF vapor etching residuals, the released devices were actuated using an electrical probing station with needle probes in contact with the aluminum areas and the silicon substrate. A DC bias from 0 V to 40 V was applied, whereas the capacitance was simultaneously measured with AC probing at 10 kHz with an oscillation amplitude of 100 mV.

### 2.6. Finite Element Model

The mirrors were modeled using the finite element method with Ansys™ software. A coupled field electrostatic analysis was performed using the Ansys Maxwell module. Before fabrication, the model of the desired mirrors was used to assess the expected performance in terms of actuation voltage and resonance frequency, providing input for the design phase. After fabrication, the actual model of the fabricated mirrors was also created and analyzed. In this second model, the following non-idealities were considered:

- silicon nitride final thickness: as the HF vapor etching has a non-negligible etch rate of silicon nitrides, the final thickness of the silicon nitride is less than the nominal thickness;
- HF vapor residuals: the residuals’ height and rigidity can reduce the mirror movement range;
- mirror radius: an uncertainty in the sacrificial etching of silicon oxide determines a difference between the nominal and actual mirror radius, this having a major effect on its mechanical behavior;
- stress of the aluminum film: this value depends on the aluminum thickness, deposition method and thermal load of the specific fabrication process and, like mirror radius, it has an important role in the mechanical performance.

The simulation results (*C_mir_*) were multiplied with the number of the fabricated mirrors (*N*), and the constant capacitance *C*_0_ was added, generated by the static areas of the device. This allows the correlation of the experimentally measured mirror device total capacitance (*C_dev_*) with the applied voltage (*V*), in accordance with Equation (1).

*C*_*dev*(*V*)_ = *C*_0_ + *NC*_*mir*(*V*)_ (1)

### 2.7. Optical Characterization

**Figure 4:**
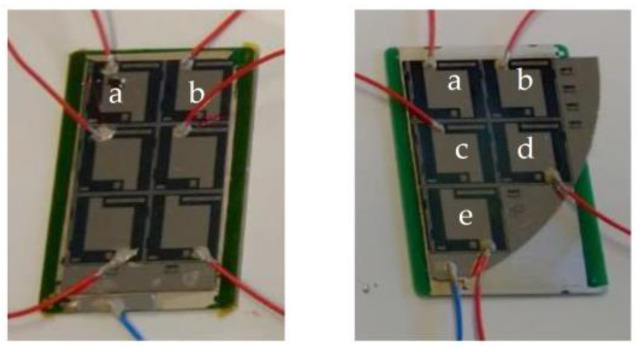
Wiring of the mirror prototypes for the optical testing. Sample 1 (**left**) and 2 (**right**). Letters indicate individual mirrors identifiers of the devices that were optically characterized.

To enable the characterization, a matrix of mirror devices of each sample was separated from the wafer by mechanical cleaving along the silicon crystal planes. The wafer portions were subsequently glued to a PCB substrate, and the wafer backside was electrically connected with the front using conductive paste. The mirrors were wired as reported in Figure 4. They were connected in parallel to the positive terminal of a voltage generator (red wires); each active submirror was glued to one end of the corresponding red wire with conductive liquid adhesive. The blue wire was glued to the common contact of the chip and connected to the ground of a voltage generator.

A preliminary assessment of the optical features was carried out at the SCF_Lab, a credited testing laboratory for space grade mirror certification. In the international framework of mirrors qualification for aerospace, SCF_Lab has developed the concurrent measure and modeling of CCR’s optical FFDP (far field diffraction pattern), and temperature distribution of laser ranging LRAs (laser retroreflector arrays) in a laboratory-simulated space environment with respect to temperature, vacuum, and solar constant using the AM_0_ solar simulator [13].

Measurements were performed in the near-field regime, through a 4D Technology AccuFiz wavefront Fizeau interferometer, using in-house developed and manufactured optics.

The near-field campaign consisted of reflected wavefront (wv) characteristics measurements. Using the Fizeau interferometer, it is possible to measure the near-field wavefront reflected by the mirror under investigation, which brings with it the optical “fingerprints” of the tested mirror itself. The comparison between the ideal plane wavefront emitted by the interferometer and the “slightly” aberrated one retroreflected by the mirror allows for collecting information about the quality of optical surfaces. The MEMS mirror-reflected wavefront characteristics were measured with and without the applied voltage to assess the effects of mirror actuation.

## 3. Results

Two sets of mirrors (samples 1 and 2) were manufactured and tested. Sample 1 mirrors had a target radius of 35 μm at a 70 μm pitch, and sample 2 mirrors had a target radius of 45 μm at a 90 μm pitch.

### 3.1. Morphological Characterization

The fabricated devices were first characterized in terms of their effective dimensions. In the areas where silicon nitride was fully exposed to HF vapor etching, a silicon nitride thickness of 60 nm was measured, compared to the initial deposited thickness of 192 nm. This indicates a silicon nitride etch rate of 5.6 nm/min during HF vapor release.

**Figure 5:**
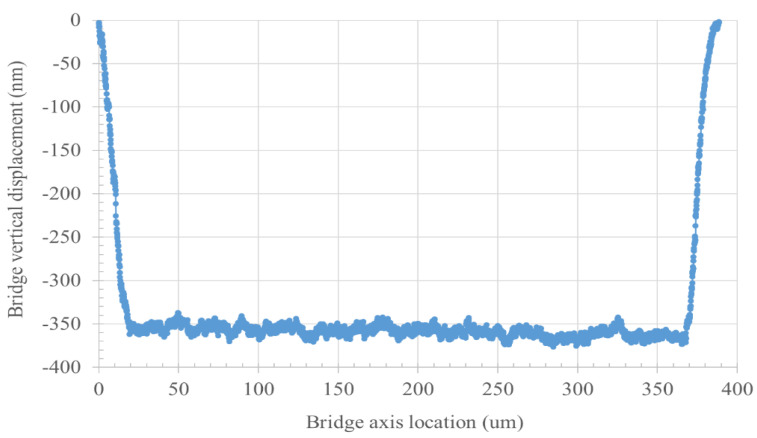
mechanical profile of a bent aluminum bridge.

Residuals of HF vapor etching (Figure 3) tend to deform under the direct load of a mechanical profilometer stylus; therefore, they were indirectly measured by profiling suspended bridges that distribute the profilometer load evenly on the underlying residuals, thus avoiding their compression. An example of mechanical profiling is shown in Figure 5. The expected displacement is the sum of sacrificial silicon oxide thickness and etched silicon nitride thickness, which is 532 nm (400 nm of SiO + etchied SiN gap of 132 nm), whereas the measured displacement is approximately 350 nm. The difference between the two provides an estimate of the HF vapor etching residual peak thickness of approximately 180 nm.

**Figure 6:**
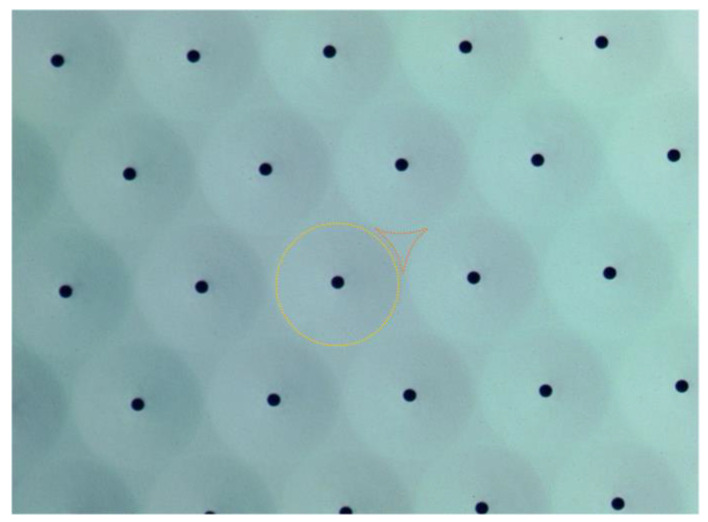
Optical image of a mirror matrix (sample 1) with grazing illumination to highlight the mirror effective area (yellow) and the oxide supports areas (orange).

The mirrors effective radius was measured from optical images. Sample 1 mirrors (target radius of 35 μm) measured radius was 37 μm (Figure 6). Consequently, the produced mirrors in sample 1 were not perfectly circular, as an over-etch of the silicon oxide occurred, which completely removed the oxide layer that should separate the mirrors. The shape is discussed in the simulation results section.

Sample 2 mirrors (target radius of 45 μm) measured radius was 42 μm, which resulted in perfectly circular mirrors with at least 6 μm of a silicon oxide boundary between each mirror. This change in the diameter is due to the difference in the HF vapor etching rate from the nominal value, caused by specific design effects such as area loading.

### 3.2. Aluminum Residual Stress

The aluminum layer changes from a slightly compressive as-deposited stress to a tensile stress at the end of the fabrication process. The deposited aluminum stress was measured as −25 MPa (st.dev. 3 MPa), and it had a 172 MPa (st.dev. 2 MPa) residual stress after the fabrication process with equivalent thermal cycling. This result is in agreement with the literature [14] where a slightly compressive stress is reported for as-deposited aluminum thin films. It is important to note that a tensile residual stress is advisable for suspended structures, whereas compressive stress may cause buckling.

### 3.3. Electrostatic Actuation

**Figure 7:**
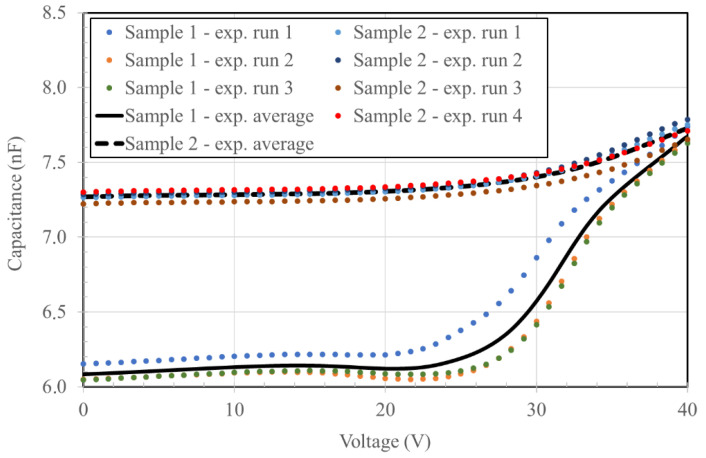
actuation voltage vs. mirror capacitance profiles.

A plot of the measured device capacitance in dependence of the actuation voltage is reported in Figure 7 for sample 1 (pitch 70 μm, radius 37 μm) and sample 2 (pitch 90 μm, radius 42 μm) devices, which consisted of 20,449 and 12,321 mirrors, respectively. The measurement was repeated several times on both devices, with the average plot presented in the same figure. Sample 1 mirrors exhibit a much more pronounced difference between the non-actuated and actuated mirrors at 40 V, which is a direct consequence of their larger active area compared to the sample 2 mirrors, due to the fully separated circular mirrors in sample 2 that will be discussed in the finite element analysis.

### 3.4. Finite Element Analysis

**Figure 8:**
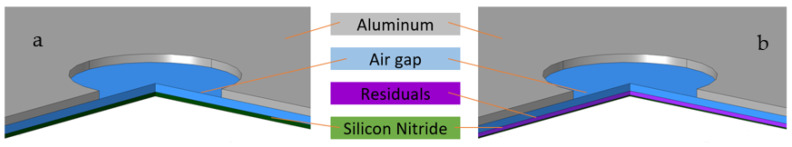
Designed and produced sizes of mirrors. (**a**) Designed mirrors: radius 35/45 μm, silicon nitride thickness = 200 nm, no HF residuals; (**b**) Fabricated mirrors: radius 37/42 μm, silicon nitride thickness = 60 nm, residuals height = 180 nm.

The designed and produced mirror dimensions are different (Figure 8), as reported in the morphological characterization. The differences are due to the formation of residuals, the thinning of the SiN layer, and the change in the mirror’s radius.

A complete set of finite element simulations was performed to model the behavior of the fabricated devices. In particular, the goal of the simulation was to obtain the effective resonance frequency and to assess the impact of fabrication parameters on the full range of actuation voltage.

**Figure 9:**
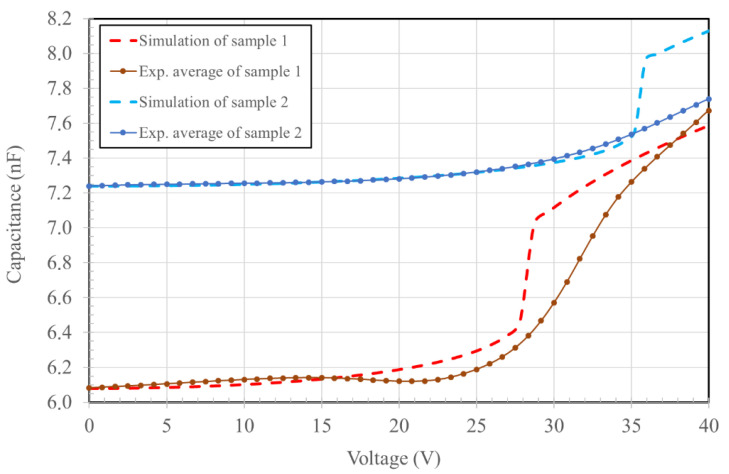
Capacitance versus applied voltage of the simulations and experimental results for the sample 1 and 2 devices.

The comparison between experimentally and numerically determined *C*_(*V*)_ for both produced samples is presented in Figure 9.

The simulation of the fabricated devices reported in Figure 9 was made using their actual parameters (mirror radius, SiN thickness, and HF residues thickness) obtained from direct measurements after fabrication (as reported in the characterization). The only parameter that was not determined by a direct measurement is the Young’s modulus of the aluminum film, which was set at a value of 69 GPa extracted from the literature [15]. Pull-in effect is visible for both simulated samples, at 28 and 35.5 V. Pull-in voltage is an indication of the full range displacement voltage, as it initiates when the mirror touches the substrate and starts collapsing on it.

**Figure 10:**
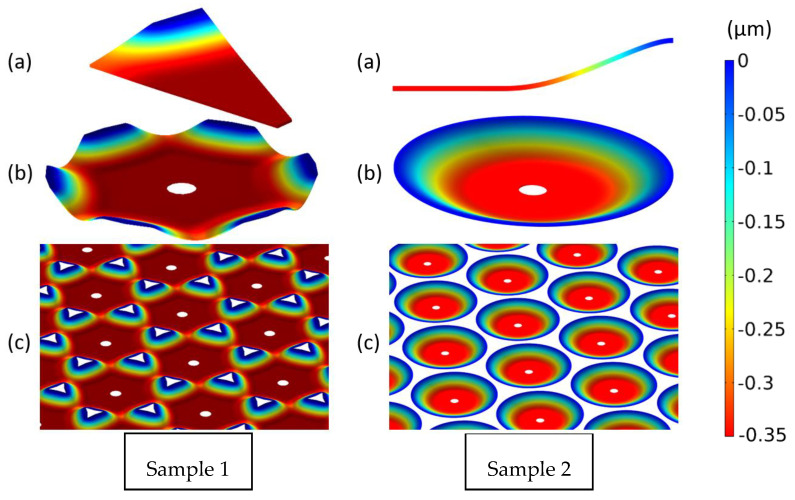
The deformation at 40 V of the sample 1 and sample 2 mirrors, (**a**) the modeled and simulated geometry, (**b**) a single mirror, and (**c**) an array of mirrors.

The simulated mirror deformation at 40 V (fully collapsed) is reported in Figure 10. The numerical computation of sample 1 required a 3D simulation, as the produced mirrors were not a perfect circular shape, whereas the sample 2 mirrors could be simulated in 2D. The shape of sample 1 actuated mirrors, due to excessive removal of the underlying silicon oxide, is reported in Figure 10b. The active surface of the sample 1 device is much larger compared to the sample 2 device, which is also confirmed by the larger difference in capacitance between the initial state and after the pull-in deformation.

The obtained finite element model was used for eigenfrequency analysis, as the natural frequency is the limiting factor for the velocity of actuation and therefore for data transfer speed in free-space optics applications. The derived natural frequency was 3.5 MHz for sample 1 mirrors and 2.5 MHz for sample 2 mirrors.

Further, finite element simulation was used to assess the effect of the main variables. We focused on the parameters which are prone to changes due to the fabrication process: E_alu_—Young’s modulus of aluminum, S_alu_—prestress of the aluminum, R_mir_—radius of the mirrors, and H_SiN_—thickness of the silicon nitride layer. In the case of the geometrical parameters, change is due to pattern transfer non-idealities typical of the microfabrication process. In the case of the aluminum elastic modulus, changes may occur as the film thickness is changed (particularly if decreased), due to the presence of native oxide on both sides of the aluminum thin film which forms upon exposure to air.

**Figure 11:**
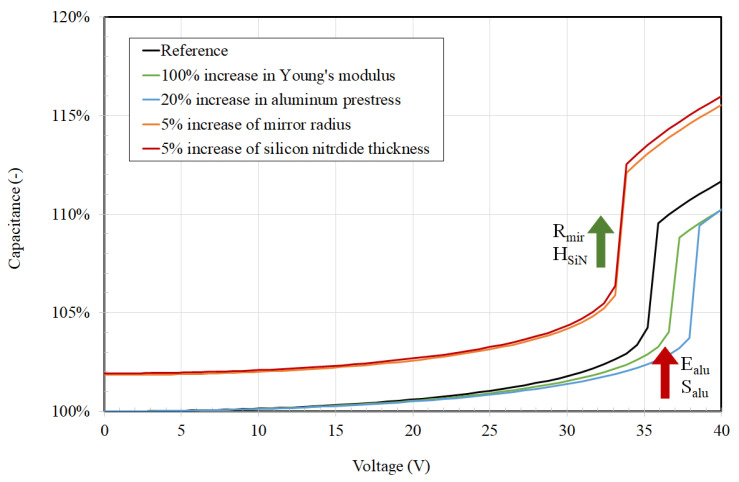
Effects of multiple parameters on the capacitance.

Indeed, at film thickness of a few 100 nm, the native aluminum oxide contribution becomes important and may affect the overall elasticity of the mirror. An example of simulation results is reported in Figure 11. The presented reference curve is given for the fabricated mirrors of 42 μm, SiN thickness of 60 nm, aluminum prestress of 172 MPa, and the nominal Young modulus of aluminum. The parameter sensitivity of the mirror movement (capacitance) is presented in Figure 11 by varying four parameters. The most influential parameters are the mirror radius and the thickness of the silicon nitride. For both, an increase of 5% causes a change in the pull-in voltage from the reference 35 V to 33 V. A 100% increase in the Young’s modulus has a smaller impact, with the pull-in voltage rising to 36.5 V. The last of the four analyzed parameters was the aluminum prestress, which we increased by 20%, causing a pull-in voltage increase to 38 V. All of the simulated actuations of the mirrors were performed by increasing only one parameter, while all of the remaining initial and boundary conditions remained the same.

### 3.5. Optical Performance

A matrix of mirror devices of each sample was prepared and wired as reported in Figure 4 for the optical characterization.

Due to alignment issues, the measurements of sample 1 were performed on the “top” two mirrors only (identified as a and b, from left to right). Measurements of sample 2 were performed on all five submirrors. Sample 1 was actuated at 35 V, whereas sample 2 had current discharges above 5 V, which made it impossible to fully actuate the mirrors. As the devices were individually tested before assembly on the support PCB and soldering, these discharges are to be attributed to the assembling on PCB substrates.

**Figure 12:**
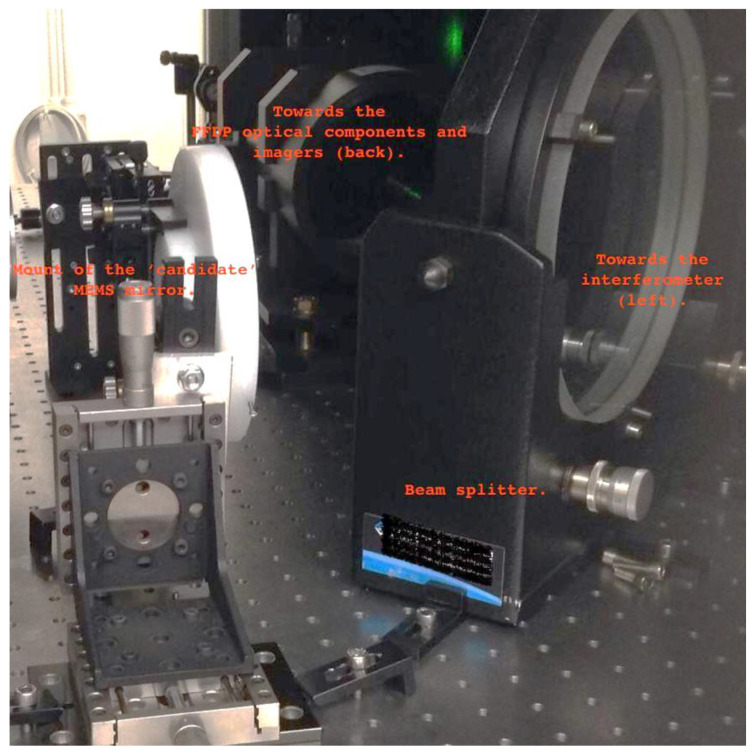
Excerpt of the mirror positioning on the optical bench.

**Figure 13:**
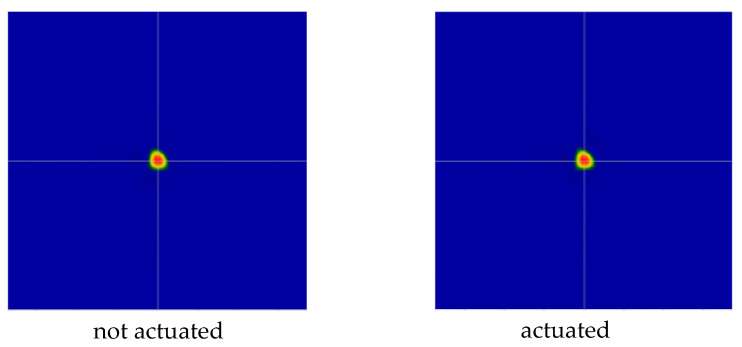
As an example, 2 “qualitative” FFDPs of sample 1, mirror b, computed through the interferometer software in arbitrary units, at the operational wavelength of 633 nm.

**Figure 14:**
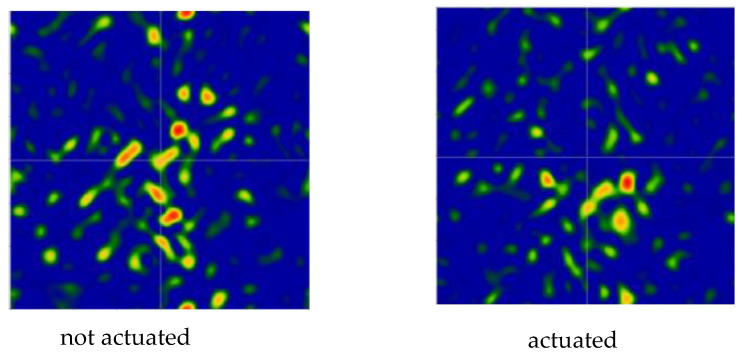
As an example, 2 “qualitative” FFDPs of sample 2, mirror d, computed through the interferometer software in arbitrary units, at the operational wavelength of 633 nm.

Table 1 and Table 2 summarize the measured mirror sample optical features. Figure 12 shows an excerpt of the mirror positioning on the optical bench. For each device we analyzed an interferogram produced by averaging over 40 shots. We extracted the following information for each mirror at the operational wavelength of the interferometer (633 nm):

- The PV (peak-to-valley) error, which is measured comparing the reflected wavefront with the non-aberrated one emitted by the interferometer itself (Table 1 and Table 2, Columns 3 and 5). This quantity provides information about the “worst” possible waviness of the surface of interest, averaged out over the whole analyzed area. The associated measurement error is ± 0.01 wv;
- The RMS (root mean square) error, which is similar to the PV, but it provides information about how smooth a wavefront is on average and “locally”, in a statistical sense (Table 1 and Table 2, Columns 4 and 6). The associated measurement error is ± 0.01 wv;
- A “qualitative” FFDP, computed through the interferometer software in arbitrary units (Figure 13 and Figure 14).

Figure 13 and Figure 14 report the FFDP images of both samples, with and without electrostatic actuation. For sample 1 (Figure 13), without activating the surfaces (left) the diffraction pattern closely resembles that of an “ideal” (square) opening; even with the applied voltage (right), and hence activating the surfaces, the diffraction pattern closely resembles that of an “ideal” (square) opening.

Differently, in the case of sample 2, without activating the surfaces (Figure 14 left), the diffraction pattern is “scrambled” as expected looking at the quantitative measure of PV and RMS as per the Table; even with the applied tension, and hence activating the surfaces, the diffraction does not improve (Figure 14 right).

## 4. Discussion

The difference between chip actuation profiles reported in Figure 7 can be attributed to localized morphological defects. Due to the large area of each mirror and the very large number of circular membranes in each mirror, a fluctuation is expected because of point defects. Further, it can also come from poorly controlled environmental conditions, as these devices are exposed to unfiltered air during electrical testing. At this stage, the process yield has not yet been assessed, and it will be determined once a sufficient number of devices is completed.

The differences between the simulated and experimental results (Figure 9) become pronounced in the vicinity of the pull-in voltage, which is the voltage at which the mirror is subjected to a sufficient force to overcome the aluminum layer tension and collapse on the bottom layer (HF residuals in our case). The pull-in step is not observed experimentally. This could be explained by two factors:

- polymer residuals may act as a soft surface on which the mirror lands with a discharge effect due to the physical contact between the metal plate and the dielectric polymer;
- the very large number of mirrors that are simultaneously actuated in the experiment may smooth the transition to pull-in actuation. As local morphological differences may occur from the edge to the center of the chip, the single mirrors are expected to enter the pull-in phase at slightly different voltages, thus smoothing the overall pull-in step. On the contrary, in the numerical results, we assumed all of the mirror sizes and boundary conditions are perfectly identical.

The numerical simulations are coherent with the experimental results before and after the pull-in voltage, which suggests that the pull-in phenomena is prevented in the real devices, but the overall behavior still follows what is expected from the finite element model.

As an estimate of the mechanical actuation voltage, pull-in can be considered as a reference that indicates the required voltage to bring the central part of the mirror in touch with the substrate.

Indeed, the optical actuation voltage is expected to be less. A very small mirror deflection of a fraction of degree may provide the necessary divergence of the reflected laser beam, depending on the mirror setup and receiver distance.

By changing the model parameters in the defined ranges (Figure 11), we see that the pull-in voltage changes from 33 to 38 V, which indicates that a small voltage correction can compensate for fabrication fluctuations and non-idealities. This suggests that the present approach is robust towards microfabrication-related variations.

Further, despite the low actuation voltage of 5 V, sample 2 exhibited an FFDP pattern change indicating the mirror is optically actuated, as can be seen in Figure 14. Differently, due to the perfect centering of the FFDP pattern in sample 1, no difference is observed between the actuated and unactuated mirror state, despite the higher voltage.

Putting together the manufacturing information with the measured data of Table 1 and Table 2, and Figure 13 and Figure 14, there seems to be a fabrication-dependent waviness. Sample 1’s average PV @ 0 V is 2.27 wv, whereas sample 2’s average PV @ 0 V is 5.81 wv; sample 1’s average RMS @ 0 V is 0.37 wv, whereas sample 2’s average PV @ 0 V is 0.60 wv. This suggests that the mirror quality may be related to the mirror diameter, with larger diameters being more prone to surface degradation.

Further comparing the two mirrors, it is possible to see a clear relationship between the FFDP “quality” and surface roughness, with or without provision of electrical potential. The larger the PV and RMS errors (as per Table 1 and Table 2), the farther away we get from an “ideal” distribution of the diffraction pattern. When the PV error is of the order of 10 wv or more, the diffraction pattern is compromised, and the reflected photons are scattered all over the detector area and beyond. 

The large range of roughness values on sample 2 clearly indicates that the fabrication and testing process stability has to be improved, in terms of the following:

- defects that may alter the behavior of a single mirror, given the large device area, possibly coming from the environment (particles) outside the microfabrication cleanroom;
- hard baking processes uniformity, which greatly affects the aluminum surface quality.

## 5. Conclusions

The design, fabrication, and characterization of a micro electromechanical (MEMS) mirror with capacitive actuation for free space optics communication is reported. Thanks to a novel fabrication sequence, the present mirror device has lower actuation voltage (<40 V) and higher operational frequency than those from previous articles, with a natural frequency above 2 MHz. This makes it compatible with very high frequency free space optics applications onboard space vehicles, where the available voltage is limited. The present mirror material is also compatible with space applications due to the absence of gold on the reflecting areas. The device was characterized, and its main parameters were obtained from experimental data combined with finite element analysis, enabling future design and fabrication improvements of the reported MEMS technology. By optical characterization of the far field diffraction pattern, good performance was observed, while a degradation of the optical performance was assessed corresponding to degrading mirror morphology. An insight was obtained on this novel device and fabrication sequence, highlighting possible weak points and providing a preliminary assessment towards the optimization of this technology.

Future work will focus on the process and assembly yield improvement. Testing with different mirror radius and oxide thickness will be performed to explore the correlation with surface optical quality. In addition, dynamic optical testing will be carried out towards the use of this device in free space optics applications.

## Figures and Tables

**Table 1:** Measured sample 1 mirror sample optical features.

Sample ^1^	Mirror Identifier	PV@0 V [wv]	RMS@0 V [wv]	PV@35 V [wv]	RMS@35 V [wv]
1	*a*	4.03	0.65	2.48	0.40
	*b*	0.51	0.09	0.60	0.10

^1^ Sample 1 totals six mirrors: due to alignment issues, measurements were performed on the “top” two mirrors only (identified as a and b, from left to right as reported in Figure 4).

**Table 2:** Measured Sample 2 mirror sample optical features.

Sample ^1^	Mirror Identifier	PV@0 V [wv]	RMS@0 V [wv]	PV@5 V [wv]	RMS@5 V [wv]
2	*a*	5.10	0.51	5.87	0.60
	*b*	7.85	0.67	8.38	0.59
	*c*	1.99	0.22	3.09	0.27
	*d*	12.79	1.44	10.52	1.27
	*e*	1.32	0.18	1.35	0.15

^1^ Sample 2 totals five mirrors: measurements were performed on all the five mirrors (identified as *a* to *e*, from left to right, from top to bottom as reported in Figure 4).

## Data Availability

Not applicable.
